# Antitumor efficacy of D2C7-(scdsFv)-PE38KDEL, a novel immunotoxin targeting EGFRwt and EGFRvIII, by convection-enhanced delivery in orthotopic brain tumor mouse models

**DOI:** 10.1186/2051-1426-1-S1-P126

**Published:** 2013-11-07

**Authors:** Xuhui Bao, Vidyalakshmi Chandramohan, Stephen T Keir, Charles N Pegram, Roger E McLendon, Chien-Tsun Kuan, Ira H Pastan, Darell D Bigner

**Affiliations:** 1 Pathology, Preston Robert Tisch Brain Tumor Center, Durham, NC, USA; 2 Neurosurgery, Huashan Hospital, Fudan University, Shanghai, China; 3 Laboratory of Molecular Biology, Center for Cancer Research, National Cancer Institute, National Institutes of Health, Bethesda, MD, USA

## Objective

**Figure 1:**
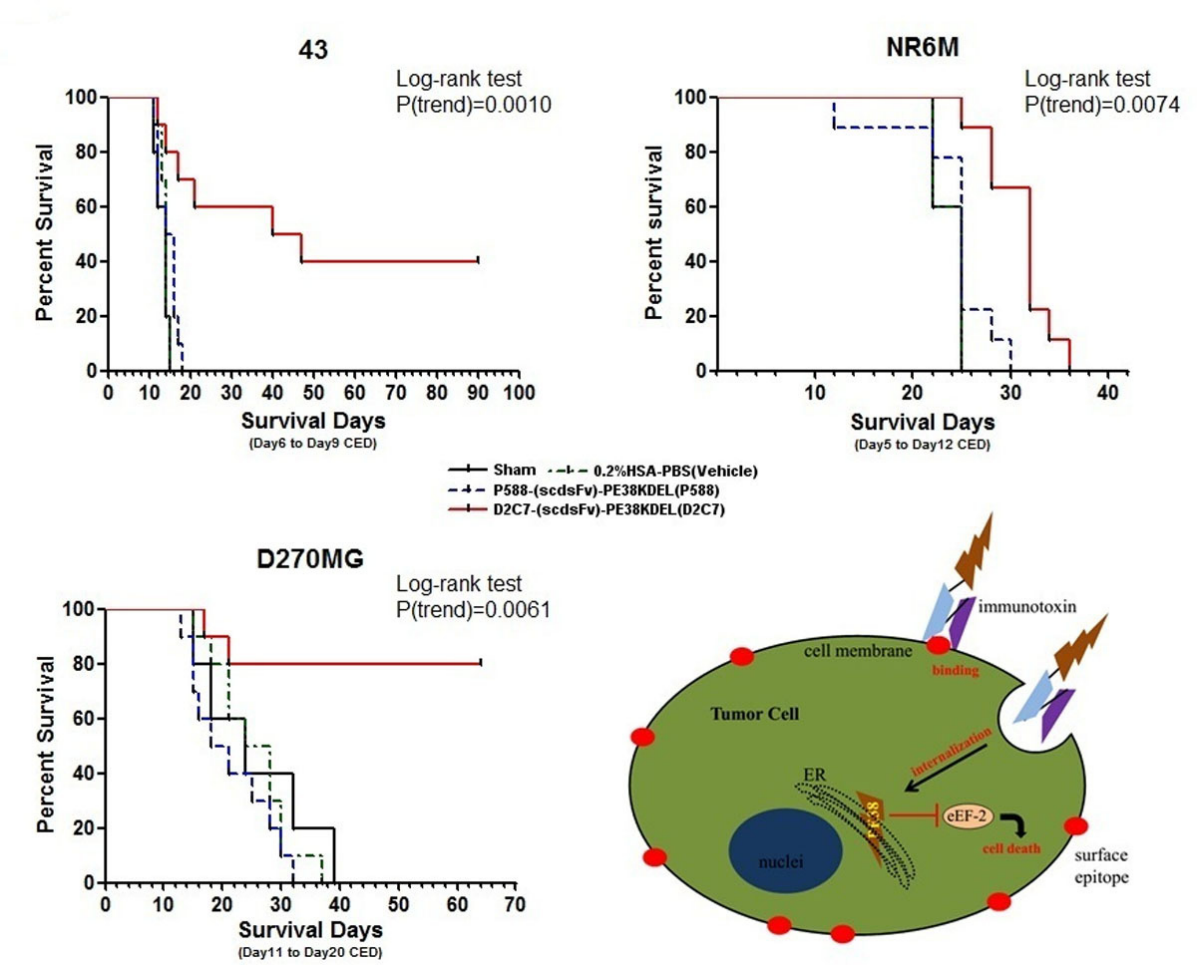
Kaplan-Meier survival curves of different brain tumor models and the immunotoxin killing paradigm

The epidermal growth factor receptor (EGFR) gene is most frequently amplified and overexpressed, along with its truncated mutant, EGFRvIII, in glioblastomas. We tested the antitumor efficacy of the recombinant immunotoxin, D2C7-(scdsFv)-PE38KDEL (D2C7-IT), which is reactive with a 55-amino acid (AA) region present in the extracellular domain of both EGFRwt and EGFRvIII proteins (Figure 1), by convection-enhanced delivery (CED) in orthotopic brain tumor mouse models established with human glioblastoma xenograft cells.

## Methods

Orthotopic brain tumor models were established by inoculating 43 (EGFRwt expressing glioma cell), D270MG (EGFRwt and EGFRvIII espressing glioma cells), and NR6M (EGFRvIII expressing fibroblast cells) intracranially in the immunocompromised mice. CED was achieved by inserting a cannula into the brain tumor site, which in turn was connected to a subcutaneous osmotic pump delivering the immunotoxin into the tumor microenvironment. The antitumor efficacy was evaluated by Kaplan-Meier survival analysis.

## Results

In the orthotopic brain tumor models of 43, NR6M, and D270MG, D2C7-IT therapy via CED significantly prolonged the median survival time (MST) of the treatment group by about 1 month (P=0.0010), 1 week (P=0.0074), and over 1 month (P=0.0061), respectively, compared with that of vehicle or negative control groups (Table 1, Figure 1).

**Table 1:** Comparison of MST among different groups in three xenograft mouse models

(Day)	Vehicle	P588	D2C7	Log-rank test
43 MST	14	15	43.5*	0.0010
NR6M MST	25	25	32	0.0074
D270MG MST	26	19.5	64**	0.0061

*Last 4 mice were euthanized on Day 90;

**Last 8 mice were euthanized on Day 64.

## Conclusion

In the orthotopic brain tumor mouse models, the D2C7-IT therapy via CED exhibited a robust therapeutic potential in treating brain tumors expressing EGFRwt, EGFRvIII, and both EGFRwt and EGFRvIII.

